# “I have to provide for another life emotionally, physically and financially”: understanding pregnancy, motherhood and the future aspirations of adolescent mothers in KwaZulu-Natal South, Africa

**DOI:** 10.1186/s12884-020-03319-7

**Published:** 2020-10-14

**Authors:** Desiree Govender, Saloshni Naidoo, Myra Taylor

**Affiliations:** 1 KwaZulu-Natal Department of Health, Durban, South Africa; 2 School of Nursing and Public Health, Discipline of Public Health Medicine, University of KwaZulu-Natal, Durban, South Africa; 3 Developing Research Innovation Localisation and Leadership (DRILL) Fellow, Durban, South Africa

**Keywords:** South Africa, Adolescent pregnancy, Adolescent motherhood, Psychological distress, Healthcare services, Sexual and reproductive health

## Abstract

**Background:**

Adolescent pregnancy and motherhood has been a controversial and much disputed subject within the field of public health. Early childbearing is not only characterized as a physical body experience but also embodies the experiences and perceptions of the social norms, discourses, conflict and moral judgement. There is an increasing concern that the psychosocial challenges facing adolescent mothers remains in the background since research in this field has mainly focused on the medical and physical complications of early childbearing. The aim of this qualitative study was to explore adolescent pregnancy and motherhood in order to understand this phenomenon from the perspective of adolescent mothers and to gain insight into their future aspirations.

**Methods:**

This descriptive qualitative study was based on data from four focus group discussions with adolescent mothers utilising healthcare services at a district hospital in Ugu district, KwaZulu Natal, South Africa. The data were audio-recorded and transcribed verbatim, then analysed using thematic analysis.

**Results:**

Some adolescent mothers’ partners were in denial and rejected them and the child while others’ partners were happy and supported them during their pregnancy. Families’ reactions to the pregnancies ranged between anger and disappointment to abandonment, the silent treatment, and acceptance and forgiveness. The psychological issues experienced by some of the adolescent mothers included suicidal ideation, guilt, loneliness, anxiety, and stress. They also experienced financial constraints, difficulty in returning to school, and stigmatisation in society. The participants envisioned completing their education, focusing on their dream careers, and contributing positively to society.

**Conclusion:**

Experiences of adolescent pregnancy and parenting are multifaceted and the healthcare needs of pregnant and parenting adolescents extend beyond information and knowledge. A multidisciplinary approach is required in the care of adolescent mothers. A key policy priority should encompass the collaboration of different professionals from various healthcare sectors to assist adolescent mothers in achieving better health and psychosocial and socio-economic outcomes as steps to securing a better future for them.

## Background

To date, adolescent pregnancy and parenting has been a controversial and much disputed subject in the public health field [1]. Adolescent pregnancy and childbearing have been debated as a “crisis” with the views that adolescent mothers experience negative health, birth, economic and social outcomes [2]. However, the opposing view is that the obstetrical risks associated with adolescent pregnancy and childbearing is attributed to adverse social and economic factors rather than the chronological age [3]. The feminist perspective argues that adolescent pregnancy and childbearing can be a positive experience and the only crisis is the lack of support available to young mothers [2, 3]. Furthermore, Rock [2] describes adolescent mothers as maternal beings that are capable of loving and nurturing their children. Adolescent childbearing has been accepted by traditional societies as normal reproductive behaviour [3]. The literature suggests that moral judgement and the scientific scrutiny of adolescent childbearing continue to receive attention, whereas the psychosocial experiences of adolescent mothers are often marginalised [4]. Early childbearing is much more than a physical experience as it embodies the experiences and perceptions of the social norms, discourses, conflict and moral judgement [1]. Furthermore, early childbearing is subjected to the repressive nature of medicalisation which results in the marginalisation of the transition from pregnancy to the postpartum period and motherhood.

The issue of early childbearing has been avidly discussed within the complexities of the socio-ecological system [5, 6]. The social circumstances, environment and life experiences also shapes the construction of motherhood. Adolescent motherhood is constructed within a social class domain that is subjected to the labels of ‘good’ and ‘bad’ mother [1]. Discourses on the “good mother” versus the “bad mother” is shaped by the woman’s age, her educational status, her employment status, her marital status, social assistance, the health and wellbeing, cognitive and behavioural problems of her children [2]. A primary concern is that the stigma may have a disempowering effect on adolescent mothers [7]. Pregnant adolescents are also more likely to experience poor medical and psychosocial outcomes [8].

Research on adolescent health has a tendency to focus mainly on the biological and reproductive health aspects rather than the psychosocial dimensions. The transition into motherhood can be a turbulent time for adolescent girls as they struggle with conflicting identities of being an adolescent, a mother and an adult [9]. The adolescent mother has numerous parental responsibilities as well as taking care of her own educational and developmental needs [10]. Adolescent parenthood has been associated with school dropout, unemployment, poverty and poor parenting outcomes [8].

In Sub-Saharan Africa (SSA), 50% of women living in rural areas aged 15–24 experienced a pregnancy before the age of 18 [11]. Stigma, gender inequality, gender based violence, HIV, discriminatory laws and poverty compounds the psychosocial, health and educational problems of pregnant adolescents and adolescent mothers in SSA [11]. The psychosocial and health burden of adolescent pregnancies is underscored in SSA and low and middle income countries [11]. The prevalence of adolescent childbearing in South Africa is relatively high. In 2017, approximately, 97,143 adolescent girls gave birth in South Africa which accounted for 13.9% of registered births [12]. South Africa remains largely a patriarchal society in which gender and stereotyping largely dominates the landscape of adolescent childbearing [7, 13, 14].

Earlier research in South Africa focused on the physical health outcomes of adolescent pregnancy while the focus on the experiences of adolescent pregnancy and motherhood was limited. There is an increasing concern that the challenges facing adolescent mothers remains in the background as research in this field has mainly focused on the medical complications of early childbearing [15]. It was against this backdrop that the purpose of this qualitative study was to explore and understand the phenomenon of adolescent pregnancy and motherhood and to gain insight into the future aspirations of adolescent mothers. This is a critically important study because so much of the research on adolescent mothers is judgmental, or is simply about maternal and infant health outcomes. The main integrated research questions that needed to be addressed were the following:

**(1) What are the experiences of adolescents during pregnancy and motherhood?; (2) What are the problems and needs of adolescent mothers?; and (3) What are the future aspirations of adolescent mothers?**

## Methods

### Study design

This was a descriptive qualitative study that formed part of a larger mixed-methods action research (MMAR) PhD project. The larger study aimed to develop a community of practice model for a multidisciplinary and comprehensive approach towards caring for parenting adolescent mothers. It is desirable practice that a research project is embedded within an existing paradigm. A paradigm is “an assembly of ideas that influences and guides the way researchers think about a subject” [16] (p.15). There is generally a choice of four research paradigms, namely: (1) positivism; (2) constructivism; (3) advocacy/participatory; and (4) pragmatism. This study design was framed within the pragmatic paradigm. Pragmatism is a worldview that encompasses the following elements: (1) consequences of action; (2) problem centred; (3) pluralistic; and (4) real world practice oriented. Moreover, this approach is not a slave to any one system of philosophy [17], but embraces mixed methods research. Mixed methods research has its philosophical origins in pragmatism [18] and thus a parallel can be drawn between pragmatism and a mixed methods approach to research [17]. Mixed methods researchers do not restrict themselves to one system of assumptions; rather, they are drawn to both quantitative and qualitative approaches [17]. Pragmatism is concerned with how the research question will drive the inquiry and, in this regard, the research question is more important than the methods [18]. Furthermore, pragmatism is a search for the truth and mixed methods research is often the practical solution to answering questions that cannot be answered by other worldviews [17, 18].

Pragmatism also encompasses action, knowledge and change which is located in action research [19]. Action is central in pragmatism. The qualitative inquiry in this study was to create knowledge in the interest of transformation and improvement. The stance of the pragmatist philosophy is that human actions cannot be disconnected from past experiences [19]. Action research involves the detection of a socially situated problem and the appropriate action to address the problem. The pragmatist methodological approach clears the path for problem solving which brings together quantitative and qualitative research [19].

The quantitative component of the MMAR study involved a questionnaire survey about adolescent pregnancy and sexual and reproductive health among 326 adolescents using maternal health services at a hospital in the Ugu district in KwaZulu-Natal, South Africa. The quantitative survey was also conducted to determine the prevalence rate of adolescent repeat pregnancy and its association with the socio-demographic characteristics of adolescent girls. The knowledge of reproductive and sexual health practices (e.g. contraception, HIV/AIDS, sexual risk behaviour/prevention and pregnancy) were also investigated. The survey also included questions related to current pregnancy health practices and social support for adolescent girls. The qualitative research design allowed the participants to identify with their own meanings of their social world and it provided them an opportunity to let their voices be heard about their experiences and needs [20]. The qualitative component of the study also established a portal for a better understanding of adolescent pregnancy and motherhood as well as the future aspirations of these mothers.

### Research setting

The research was conducted in a district hospital in the Ugu, southern KwaZulu-Natal. Ugu district has been classified as 84% rural while the remaining 16% is urban. The population size of Ugu district was about 722,484 at the time of the study. This district experiences the triple burdens of unemployment, poverty and inequality. The district hospital offers both antenatal and postnatal care as well as labour and delivery services. The staffing at the district hospital includes: advanced midwives, midwives, enrolled nurses, nursing assistants, social workers, dieticians, full time medical officers, physiotherapists, occupational therapists, speech language therapists and clinical psychologists. Prior to the study, the adolescent delivery rate at this district hospital was approximately 23% [21], while the prevalence of adolescent repeat pregnancy was 19.9% [22].

### The study population

The population of interest was parenting adolescent mothers (i.e., first time and repeat mothers) in the age group 13 to 19 years. The definition of a first time adolescent mother that applied to this study was a female between 13 and 19 years of age who had given birth for the first time to a live infant, while the definition of adolescent repeat mothers was female individuals between 13 and 19 years of age who had given birth twice or more to live infants.

### Sampling method

The quantitative strand of the larger PhD study was also employed as a model for the selection of the participants of this qualitative strand. This model can be explained as a process whereby categories of first time adolescent mothers and adolescent repeat mothers identified by the quantitative data were purposefully selected for the qualitative strand [18]. The quantitative study identified participants who had experienced adolescent pregnancy and motherhood and who were willing to share their experiences. The participants were given information pamphlets about the qualitative study. Consent to participate in the focus group discussions (FGDs) were obtained for all participants over the age of 18 years and parents’/guardians’ as well as the participants’ consent was obtained for those under the age of 18 years.

### Sample size

Four focus group discussions were held. These discussions involved 18 participants in total. The minimum and maximum number of participants for the focus group discussions were 4 and 5. Data saturation was reached by the fourth focus group discussion.

### Data collection

The data for this strand of the study were collected using focus group discussions. A focus group discussion guide was designed in collaboration with a clinical psychologist, a social worker and a clinical midwife. The guide was also perused and approved by the research supervisors (SN and MT) and the Biomedical Research Ethics Committee (BREC) of the University of KwaZulu-Natal (UKZN). The discussions were conducted between March and April of 2018. The participants unanimously agreed that the focus group discussions should be conducted over weekends. Each of the focus group discussions was held in a comfortable, non-threatening and private learning resource centre at the hospital.

Group confidentiality was discussed with the participants. Consent to audio record the discussions was sought from and granted by the participants. The home language of the participants was IsiZulu and the discussions were thus conducted in this language and facilitated by the first author (DG), qualified social worker and a trained research assistant. DG is a chief physiotherapist at the district hospital. She (DG) is a clinician researcher and has experience in mixed methods research. DG attends to referrals from the maternal and child health divisions. In this regard, she (DG) has extensive insight into the care of pregnant and parenting adolescents. The advantage of being familiar with the research site and participants enhanced the relationship and communication between the clinician researcher and the participants. The participants are more likely to reveal intimate details of their lives to an individual that they are conversant with rather than strangers. In this regard, the richness and authenticity of research data is dependent on the relationship between the researcher and participants [23]. The duration of the discussions ranged from 120 to 180 min. A notebook was also used to record field notes. The fieldnotes included observations, behaviours and sensory impressions. Data were collected on the experiences of adolescent pregnancy and parenting, healthcare services, perceptions of sexual risk behaviour, and the future aspirations of the participants. The findings that are presented in this paper relate exclusively to the participants’ experiences of pregnancy and parenting and their future aspirations. A later paper will present and discuss the findings regarding the participants’ understanding of sexual risk behavior.

### Data analysis

The recordings from the focus group discussions were transcribed in verbatim format. The transcriptions were translated into English by a research assistant who was proficient in both IsiZulu and English. The anonymity of the participants was maintained by assigning a pseudonym to each. The data were analysed using the thematic analysis process as proposed by Braun and Clark [24]. The transcripts were repeatedly read to become familiar with the data, and the data were coded and then categorised into themes.

The credibility of the findings was confirmed by sharing the data and the findings with the research supervisors who ensured that the data had been analysed appropriately. The transcripts were taken back to the participants for review and reflection. The research participants were familiar to the research team which means that trust and confidence had been established prior to data collection. Clear, thick descriptions of the research process may assist future researchers with the transferability of the findings.

## Results

### Characteristics of participants

In total, 18 adolescent mothers participated in the study (Table 1). The ages of the participants ranged between 16 and 19 years. Nine of the participants had experienced repeat pregnancies while nine were first time mothers. The majority of the participants (*n* = 13) had dropped out of school. The ages of the birth fathers/partners ranged between 19 and 36 years (Table 2). The average age of the birth father/partner was found to be 24.38 years. Eight themes emerged from the data.

**Table 1:** Socio-demographic details of participants (*n* = 18)

Participant	Number of pregnancies	Pregnancy Planned/Unplanned	Living situation	Relationship status	Education level	Employment status
Participant 1	2	Unplanned	Living with biological mother	In a relationship, not living together	Junior high Dropped out of school	Unemployed
Participant 2	2	Planned	Living with biological mother	Engaged to father of children. Lobola has been paid by partner	Senior high Studying at an FET college	Unemployed.
Participant 3	2	Unplanned	Living with biological parents	In a relationship, not living together	Junior high Dropped out school	Unemployed
Participant 4	2	Unplanned	Living with biological mother and siblings	In a relationship. not living together	Senior high Dropped out of school	Unemployed
Participant 5	2	Unplanned	Living with biological sister	In a relationship. not living together	Junior high Dropped out of school	Unemployed
Participant 6	1	Unplanned	Living with biological parents and siblings	In a relationship. not living together	Senior high	Unemployed
Participant 7	1	Unplanned	Living with an aunt.	In a relationship. not living together	Junior high Dropped out of school	Unemployed
Participant 8	1	Unplanned	Living with biological mother and siblings	In a relationship. not living together	Senior high	Unemployed
Participant 9	1	Unplanned	Living with biological mother and siblings	Single	Senior high	Unemployed
Participant 10	1	Unplanned	Living with an aunt and biological siblings	Single	Senior high Dropped out of school.	Unemployed
Participant 11	1	Unplanned	Living with sister	In a relationship. not living together	Senior high Dropped out of school	Unemployed
Participant 12	1	Unplanned	Living with biological mother	Single	Junior high Dropped out of school	Unemployed
Participant 13	1	Unplanned	Living with an aunt	In a relationship. not living together	Senior high. Dropped out of school	Unemployed
Participant 14	1	Unplanned	Living with biological sister	In a relationship. not living together	Senior high Dropped of School	Unemployed
Participant 15	2	Unplanned	Living with maternal grandparents	Single	Junior high Dropped out of School	Unemployed
Participant 16	2	Unplanned	Living with biological mother and sibling	Single	Primary school Dropped out of school	Unemployed
Participant 17	2	Unplanned	Living with maternal grandmother	In a relationship. not living together	Senior high	Unemployed Studying at University
Participant 18	2	Unplanned	Living with biological mother and siblings	In a relationship. not living together	Senior high Dropped out of school	Unemployed

**Table 2:** Age profile of the participants and their partners (birth fathers) in Kwa Zulu Natal, South Africa

Characteristics		Number of participants (Percentage)
**Age of participants**	16–17	5 (28%)
	18–19	13 (72%)
**Age of partners (birth fathers)**	19–24	11 (61%)
	25–30	6 (33%)
	31–36	1 (6%)

### The overarching themes with verbatim quotes

#### Theme 1: the complexities of adolescent pregnancy

The theme ‘complexities of adolescent pregnancy’ underscored the difficult circumstances related to adolescent pregnancy. Factors such as unplanned and imposed pregnancies, partners’ and families’ reaction to the pregnancy, and psychological issues emerged as subthemes. Some participants (adolescent mothers) experienced denial or rejection by their partners while others’ partners were happy and accepted the reality of fatherhood. The families’ reactions ranged from anger and disappointment to abandonment, the silent treatment, acceptance, and forgiveness. The participants also described experiences of psychological trauma such as suicidal ideation, guilt, and shame**.**

##### Sub-themes

**The unplanned pregnancy**

The focus group discussions revealed that some participants experienced an unplanned pregnancy. The unplanned pregnancies were a dilemma for these adolescents. The following quotes illustrates the unexpected pregnancies among adolescents:

“My pregnancy was not planned. It just happened. We previously used protection during sexual intercourse but this time we forgot” (Participant 1)

“No, I did not plan to fall pregnant. In fact, everything happened so fast. It was my first sexual experience. I became ill and my mother took me to a private doctor. At the doctor’s room, I was tested for pregnancy. The test was positive”. (Participant 8)

One adolescent mother spoke about how rebelling against her family had led to an unplanned pregnancy.

“My pregnancy was not planned. My family chased me away from home due to misbehaviour, I decided to stay at my friend’s house. At my friend’s house, I had my own freedom and no one could control me. I did as I pleased and I got pregnant” (Participant 11)

**The imposed pregnancy**

Adolescent mothers spoke about their imposed pregnancies. These imposed pregnancies were characterised by male domination, conflict, cultural and social expectations.

“I was not ready for a child but my boyfriend told me that his friends were teasing him about not having children. He wanted me to fall pregnant and give him a child. He said this would prove his manhood and this would secure our relationship” (Participant 15)

“Yes, my pregnancy was planned. We never thought that I would fall pregnant so easily. His family had paid lobola (bridal wealth) for me. They wanted a child for their son. They even took me to the hospital for a routine check-up”. (Participant 2)

“I was in a long-term steady relationship with my boyfriend. We started having sex in 2016. I had plans to further my tertiary education in Durban. My 26-year-old boyfriend became jealous and controlling. He impregnated me” (Participant 13)

**Partner’s reaction to pregnancy**

The adolescent mothers expressed that the disclosure of their pregnancy had impacted on their relationships with their partners; some positively but most negatively. The negative reactions included rejection and anger.

“The father of my baby had rejected me. He was concerned about the fact that I had a child previously from another man. He had no children and was questioning the paternity of the baby. He told me that he will wait for the baby to be born. Throughout my pregnancy I was alone.” (Participant 4)

“My boyfriend was very angry with me. He refused to talk to me. He said he was not ready for the pregnancy. He accepted the child only after she was born” (Participant 10)

“He didn’t accept responsibility for the pregnancy. He said that anyone could have impregnated me. It was a random sexual encounter and we were not in a relationship” (Participant 16)

Some adolescent mothers expressed positive experiences such as happiness and excitement among their partners during the disclosure of their pregnancy.

“My partner was very excited. He was 25 years old and wanted a child. He accepted my pregnancy” (Participant 7).

“The father of my child was happy because he wanted this child. He also felt that I would belong to him only after the child is born” (Participant 14)

**Family’s reaction to pregnancy**

The adolescent mothers expressed that the disclosure of their pregnancy had negatively impacted on their relationships with their family members. Family relationships were strained and became dysfunctional.

“My mother was initially very angry. She and my dad refused to speak to me for three months. But my mother eventually accepted the pregnancy. She gave me emotional support and took good care of me during my pregnancy. My father was continuously angry with me. He would avoid me in the house. He told my mother that he will not support me financially. He believed I was another man’s responsibility” (Participant 13)

Some adolescent mothers indicated that their mothers were initially angry but eventually supported them during the pregnancy.

“My mother was initially angry but she supported me eventually. My father stopped talking to me during the entire time of my pregnancy. He started talking to me once the damages were paid for by my boyfriend” (Participant 14).

“My family was angry and disappointed with me. My mother refused to speak to me. At a later stage, she accepted my condition and took very good care of me. My other family members also forgave me and supported me” (Participant 5)

According to the adolescent mothers, the disclosure of their pregnancy also resulted in anger and shame among family members. Some adolescent mothers were rejected by their family members. The home environment had become volatile for these adolescents.

“My family was very angry and ashamed of me. They refused to accept my pregnancy. I continued to live in the house but they refused to take care of me and the baby” (Participant 12)

“My family chased me away. They were angry and ashamed that I got pregnant. They refused to support me emotionally and financially. I went to live with my boyfriend”. (Participant 11)

**Psychological distress**

Adolescent mothers expressed that finding out about their pregnancies had an immense impact on their psychological wellbeing. The reality of being pregnant as an adolescent was distressing. Guilt, shame and suicidal ideation (emotional and mental distress) dominated the thoughts of the adolescent mothers.

“I attempted to kill myself when I found out that I was pregnant. I was scared, angry and overwhelmed. Abortion was on my mind but I was scared that my baby’s spirit would haunt me. My aunt found out that I was pregnant. After we discussed the pregnancy issue, we decided to tell my other family members.” (Participant 5)

“When I became sick in March 2017, I suspected that I might be pregnant, I attempted suicide but I failed. Thereafter I took a pregnancy test. The test was positive. I attempted suicide again. This was another failed attempt” (Participant 9)

“I felt so guilty and depressed. I had planned to commit suicide as that would have been an easier way to solve my problem. My grandmother had prevented me from ending my life. I did not know if I wanted to keep the baby or have an abortion” (Participant 15)

“I had intense feelings of guilt and shame. I had previously attended the reed dance and I was so proud of my virginity” (Participant 8)

#### Theme 2: participants’ relationship with the father of the child and father-child interaction

This theme exposed the participants’ relationship with the father of the child as well as the prevalence of father-child interaction. Various reactions emerged: the fathers were caring and supportive, some were abusive, others found it complicated, some had a non-existing relationship, others enjoyed the active involvement of the father, while others were left alone as the father of the child abandoned them.

##### Subthemes

**Caring and supportive**

Some of the adolescent mothers described the relationship with their child’s father as caring and supportive. The responses indicate that some adolescent mothers were content with the support provided by children’s fathers.

“The father of my child is very supportive and I like his attitude towards me” (Participant 2)

“He is very caring and supportive. We are in a steady relationship” (Participant 8).

**Abusive**

In contrast to Participant 2 and Participant 8, Participant 1 and Participant 3 revealed the abusive nature of their relationships with the father of their children.

“The father of my child has a bad attitude (temperamental) and is rarely supportive. He uses vulgar language and shouts at me in public” (Participant 1).

“My child’s father is aggressive towards me. The situation is worst when he is under the influence of alcohol” (Participant 3).

**It’s complicated**

Adolescent mothers described their relationships with the father of their children as complicated. Participant 4 described the father of her child as unfaithful and promiscuous. Furthermore, Participant 4 felt that she had to accept his unfaithfulness. These responses indicate that adolescent mothers faced the pressure of “complicated” relationships.

“He is very caring and loving but he is cheating on me. He is open about his other affairs with other women. I have to accept it.” (Participant 4)

“I am in a complicated relationship. The father of my child is involved with me and another woman. His other partner is my neighbor” (Participant 5)

**The non-existing relationship**

While some adolescent mothers described their relationships as complicated with the father of their children, others revealed that they had no relationships with the father of their children.

“There is no relationship between myself and the father of my child. Our relationship ended very ugly.” (Participant 10)

“I have no relationship with my child’s father. Our encounter was brief and we never kept in touch even after my pregnancy” (Participant 16)

**The actively involved father**

The description of the ‘actively involved father’ included one who regularly visits his child, cares for the child and provides financially for the child. Participant 7 pointed out that the father of her child is making an effort to actively bond with the child.

“He visits us regularly. He plays with the baby. He is also bonding with the baby. He provides for the baby financially” (Participant 7)

“The baby’s father visits us on a weekly basis and is actively involved in the baby’s life. He also accompanies us to clinic visits” (Participant 13)

**The absent father**

Some adolescent mothers described the ‘absent father’ that doesn’t acknowledge the existence of his child and provides no financial assistance. Participant 16 remarked that the father of her child has never visited the child.

“He doesn’t acknowledge his child. He has never visited the child. He doesn’t provide child support care” (Participant 16)

“He does not visit his child. He has been absent in the child’s life. He does not provide any financial assistance” (Participant 10).

#### Theme 3: the reality of life since the pregnancy and the onset of motherhood

This theme drew attention to the changes in the participants’ lives since experiencing adolescent childbearing. The participants reflected on their changing priorities, a non-existent social life, loneliness, parenting concerns, anxiety and stress, and the disruption of their schooling.

##### Subthemes

**The needs of the child/children become the first priority**

Many adolescent mothers spoke about the prioritisation their children’s needs. Participant 1 expressed that her needs were secondary to the needs of her children.

“My responsibilities include putting the needs of my children first. Their emotional and financial needs are more important to me than my own needs” (Participant 1).

“As a young mother of two children, I prioritise their needs. In the past I was always concerned about my needs. Now my children are number one on the list” (Participant 15)

“I have become matured in my thinking and I prioritise my baby’s needs. My responsibilities have increased. I have to balance taking care of my baby and attending school” (Participant 5)

**The non-existent social life and loneliness**

Some adolescent mothers felt the loss of their social life since the inception of motherhood. The lack of family support also contributed to the ‘non-existent social life’.

“I am not allowed to socialise because my family does not help me to take care of my children. I don’t have time to visit friends or go to parties. I have also been told by my family that I should not go out because I am an embarrassment” (Participant 3)

“I don’t have time for social activities. In fact, my social life is non-existent. My sisters will not assist in caring for my children if I want to go out with friends (Participant 18)

Likewise, the loss of the adolescent mothers’ social life was also accompanied by loneliness. Participant 3 revealed that despite having a large family, she felt lonely.

“I am lonely despite having a large family. I also avoid my family because we fight a lot at times” (Participant 3)

“I feel lonely and isolated. I only have my mother and twin sister. I have no contact with friends” (Participant 16)

“I feel so lonely and isolated since my pregnancy and giving birth to my child” (Participant 10)

**Disruption in schooling**

Many adolescent mothers spoke about how the pregnancy and motherhood disrupted their formal education (attendance of school). Participant 1 expressed how prioritising her children’s needs resulted in her dropping out of school.

“I am now concentrating on my children and their needs. Being a young mother has affected my studies. I chose to raise my children and I dropped out of school” (Participant 1)

“I dropped out of school during my first pregnancy. I never returned to school because it was hard to raise a baby and concentrate on school work. Besides I could not afford a nanny (Participant 4).

From Participant 12 response, it is evident that the lack of family support has contributed to adolescent mothers’ difficulty in returning to school.

“I have not been able to return to school because I don’t have any support to look after my child. I am also very demotivated” (Participant 12)

“I found it extremely difficult to return to school. I didn’t have a nanny and I did not have the family support to return to school and look after two children (Participant 3)

**Parenting concerns, anxiety and stress**

Many adolescent mothers expressed that motherhood is accompanied by anxiety and stress. The additional responsibilities of motherhood contributed to the stress and anxiety among adolescent mothers. The participants expressed various concerns regarding the raising of their children. They perceived the following as their parenting concerns: the health and well-being of their child/children, procuring baby consumables, and securing a future for their child/children.

For some adolescent mothers, the health and well-being of their children was an overwhelming concern. Motherhood and child rearing was new to most adolescent mothers and they expressed a lack of experience in caring appropriately for their children.

“I am most concerned about my child’s health and well-being. Children are fragile and as a young mother, you always wonder if are doing everything correctly. I don’t have the experiences of older mothers” (Participant 6)

“I am scared of my children becoming ill. I am always worried that I may not be taking care of their health correctly” (Participant 1).

“The health and well-being of my children is my concern. My four months old child has severe eczema and I feel so helpless to see him in pain (Participant 16).

According to some of some adolescent mothers, they were always concerned about not having enough food, nappies and clothes for their children. The financial hardships contributed to their concerns regarding baby consumables.

“I think I worry most about my not having enough food, clothes and nappies for my baby. I don’t always have enough money for baby formula. I borrow money and nappies from my neighbour” (Participant 10)

“I am always concerned about not having enough products for my baby’s needs. I often run out of money so I don’t have enough baby formula and nappies. I buy cheap nappies and this causes rash” (Participant 12)

Adolescent mothers reported that they were scared about not being able to provide for their children in the future. The fear of failure and surviving were evident in the illustrative quotes. The lack of financial and social support contributed to the fear of the future.

“I am scared that I will not satisfy my child’s needs. I feel anxious for the future and if I will be in a stable position to provide for all his development needs” (Participant 8)

“I am concerned about for the future. I don’t know if I will be able to provide a secure future for my children. I am scared that I won’t be able to meet the demands of my growing children. My partner is unemployed. My family refuses to take care of my children financially” (Participant 18)

For most adolescent mothers, stress and anxiety was a regular occurrence. The parenting concerns were engraved in the stress and anxiety that they experienced. The adolescent mothers acknowledged that they struggled with the transition from childhood to parenthood (motherhood).

“Every day, I am anxious and stressed about my children and their future. I am an adolescent mother and I struggle with all these responsibilities” (Participant 15)

“It is very stressful because I am accountable for two lives. I have to balance motherhood and trying to care for my myself” (Participant 7)

“I feel anxious most of the time. My responsibilities have increased. I feel stressed. I fear if I am raising my child well. I fear for my child’s health” (Participant 8)

“I find that life has become very stressful these days. I become worried about my future. Being a young mother is overwhelming” (Participant 4)

**Attaining the roles of a provider and nurturer**

Some adolescents described that motherhood instilled the roles of being a provider and nurturer. The additional responsibilities were the impetus that shaped the adolescent mothers as providers and nurturers which became an everyday reality.

“I am responsible for making sure my children are happy. I am also providing for their needs by earning an income in my tuckshop. I take them to the clinic when they are ill” (Participant 3).

“My responsibilities include supporting my child financially and providing him with love. I also believe that my responsibility includes his health and well-being” (Participant 6)

“I have to provide for another life emotionally, physically and financially” (Participant 12).

“I have to take important decisions concerning my child. I have to provide for her emotional and physical needs” (Participant 17).

#### Theme 4. Sources of financial and emotional support

The participants referred to various sources of financial and emotional support, and this illustrated the prevalence of a supportive network of women in the lives of adolescent mothers and their children.

##### Subthemes

**Grandmother**

Some adolescent mothers received emotional and financial support from their grandmothers. Participant 15 (18 years old) described her grandmother as a confidante with whom she could share her problems with and depend on for advice on child rearing.

“My maternal grandmother is very supportive. I can talk to her about my problems. She also buys clothes for me and my children because the child support grant is inadequate. I learnt from my grandmother on how to care for my children. She taught me about home remedies that really work. She taught how to bathe, dress and feed my children. She loves my children a lot” (Participant 15).

“My granny supports me emotionally and financially. I also use the child grants to survive. My grandmother gave me more advice than healthcare workers about how to care for my children. She tries her best to assist me” (Participant 1)

**Biological mother**

Biological mothers also assisted some of the adolescent mothers emotionally and financially. Jenny acknowledged the overwhelming support of her biological support despite her mother’s own financial difficulties.

“My mum is my biggest supporter. She tries her best to help us emotionally and financially” (Participant 4)

“My mother assists me emotionally. My mother is very kind because we live on her grant. I could not apply for child support grant because I don’t have an identity document” (Participant 16)

**Partner’s mother**

Some adolescent mothers had the emotional and financial support of their partner’s mother (child/children’s paternal grandmother). Participant 3 and Participant 18 indicated that their partners’ mothers had a sense of responsibility towards them and their grandchildren.

“My family at home do not give me any money because they consider my pregnancies to be my mistake. They do not support me emotionally as well. So my partner’s mother gives me money. She is also kind and understanding about my problems. I use the money to buy things for my tuck-shop at home. It provides me with an income” (Participant 3).

“My partner’s mother is a very giving person. She assists me financially and emotionally. She always tries to find out my needs although she is also poor” (Participant 18)

#### Theme 5. Problems experienced by adolescent mothers

The participants perceived the following to be their problems: financial constraints, difficulty accessing healthcare services, stigmatisation by healthcare workers and exposure to a judgmental society. They felt that society regarded adolescent mothers as outcasts and criminals and that their condition was ‘contagious’. They emphasised discrimination and stereotyping by society as well.

##### Subthemes

**Financial problems**

Most adolescent mothers experienced financial constraints. Motherhood intensified the financial problems of adolescents. According to Participant 10, motherhood had revealed to her the financial implications of raising a child.

“Financial issues has been a huge problem in my life. I have to ask for money. I have learnt the hard way that raising a baby is costly” (Participant 10).

“I am not financially independent. I am dependent on others financially. The child support grant is inadequate and I cannot afford to buy much for my child”. (Participant 6)

“I have financial problems. I don’t have a child support grant. I have to borrow money from friends for baby formula and clothes. I don’t have family support” (Participant 11)

**Exposed to mockery and judged by the community**

Most adolescent mothers were exposed to mockery and judgement by the community. For some adolescent mothers, the stigma and discrimination was overwhelming. The lack of support and humiliation by the community made adolescent mothers feel alienated, angry, unhappy and lonely.

“The community treats adolescent mothers differently from other adolescent girls. They want to isolate us from adolescent girls who have not fallen pregnant. They tell other adolescent girls not to be friends with adolescent mothers. They complain that we are a bad influence”. (Participant 15)

“The community members complain about adolescent mothers and say they will influence other young girls to also misbehave. They compare adolescent mothers to other girls who are not adolescent mothers. The community members ask these girls who are not adolescents mothers to not associate with adolescent mothers. The stigma is overwhelming” (Participant 10)

“The community treats teenage mothers like criminals. They are not kind and use harsh words to describe adolescent mothers” (Participant 7).

“The community isolates adolescent mothers. They don’t want adolescent mothers to socialise with other young girls because they think we are a bad influence” (Participant 12).

“The community stigmatise adolescent mothers. They embarrass young mothers by calling us names. This treatment makes me feel aggressive” (Participant 11).

“There is mixed reaction in their behaviour towards adolescent mothers. Some think that teenage mothers are a bad influence on other girls and some are supportive and kind. I was told by some community members to drop out of school because I am a failure” (Participant 6).

“The community members enjoy gossiping about adolescent mothers. They consider adolescent mothers to be irresponsible and verbalise that adolescents fall pregnant deliberately for child support grant. They don’t believe that we use injectable contraceptives” (Participant 17).

“The members of the community gossip about adolescent mothers and tarnish our image further. They call us vulgar names and treat us worse than criminals” (Participant 18).

**Healthcare services problems**

The participants acknowledged that they experienced difficulties in accessing healthcare services for themselves and their children. The common types of healthcare service problems included long distances to the clinics, high public transportation costs, lack of empathy from nursing staff, rude and disapproving staff, mistreatment and stock out of medication needed. The inappropriate behaviour of health care providers deterred adolescent mothers from wanting to use public healthcare services.

For some adolescent mothers, long distances and high transportation costs to the local clinics were barriers in accessing healthcare services. Participant 3 has to travel approximately 40 km to her local clinic. According to Participant 3, the delays in public transport results in late arrivals at the clinic which frustrates the healthcare workers. Transport costs to local clinics also compounds the financial hardships of adolescent mothers.

“The clinic is very far from my house. I have travel about 40 km to get to my clinic. This is also costing me a lot of money” (Participant 3)

“The clinic is very far from home. Taxi fares is expensive. It takes me two hours to reach the clinic. I always have to find someone to accompany me to the clinic. I was raped at the age of 10 years and since then I am scared to go to the clinic alone” (Participant 16)

“The transport is expensive to the clinic. The clinics are far and the mobile clinics are not efficient. The mobile clinics do not have enough medication” (Participant 18)

“The clinic is far from home and it takes two taxis to reach the clinic. The transportation costs are high and I borrow transport money. Transport delays causes you reach the clinic late and then nurses get angry with you” (Participant 10)

“I don’t always have money for transport. The clinic is far from my home” (Participant 17)

Adolescent mothers described the lack of empathy of nursing staff at local clinics also hindered their access to healthcare services. Participant 9 and Participant 15 reported that patients were turned away if they reached the clinic late in the day. Participant 1 felt that nursing staff discriminated against adolescent mothers.

“The nurses turn you away if you reach the clinic at midday. The give you a return date. They are also very slow at the clinic” (Participant 9)

“The nurses become aggressive at the clinics when they see adolescent mothers. They scold you. They make fun of you. The queues are long and they make us wait” (Participant 1).

“Transport delays can’t be controlled. We arrive late at the clinic and the nurses shout at patients even if it’s not their fault. This makes me so scared to even go to the clinics” (Participant 15)

In this study, adolescent mothers described that nurses and doctors were disapproving and rude to them. The quote by Participant 6 (16 years old) illustrates that adolescent mothers do not want to be judged by healthcare providers.

“The nurses do not approve of adolescent mothers. They think we are a burden to the healthcare system. They embarrass us in the queue. The nurses even gossip with each other about us” (Participant 15)

“Doctors and nurses shout at adolescent mothers and complain that we are irresponsible having children at a young age” (Participant 16)

“The nurses embarrass us when we are sitting in the queue and also pinpoint us as adolescent mothers. They are rude when they speak to us. I feel sad because I want their help and not their judgement” (Participant 6)

For some adolescent mothers, their encounters with healthcare providers resulted in mistreatment.

Participant 4 described her negative experiences with nurses during antenatal care, labour and immunization of her child.

“Nurses are not friendly. They treat you badly during labour because they think adolescent mothers are bad. In antenatal clinics, we are scared sitting with older mothers. We know that nurses will shout at us. They also shout at us when we take our babies for immunization. I feel that they are rough with our babies” (Participant 4)

“The nurses treated me badly during my pregnancy. They embarrassed me at my first antenatal visit. The doctors can also be judgmental. They say things like adolescent mothers are very proud to fall pregnant at a young age” (Participant 1)

“Nurses at the clinic treated me badly. If they don’t have the medication we need, they tell us to buy with our own money. I once told the nurse I don’t have money to buy the medications. She asked me that why did I have a baby if I don’t have money” (Participant 12)

“I feel mistreated. Nurses shout at younger mothers. The nurses say that they don’t have medication for the babies and they refuse to assist you if you missed the immunisation date” (Participant 13)

Adolescent mothers reported that local clinics often experience stock-out of medication and this also poses as a barrier to accessing healthcare services for their children. Participant 13 expressed her disappointment in travelling long distances to the clinic and then finding out that there is no medication available for the child’s treatment.

“Sometimes the clinic does not provide the medication. They tell you to use home remedies” (Participant 6)

“It’s difficult to get to the clinic and it becomes disappointing when they don’t have medication for your child. They give you advice on home remedies” (Participant 13)

“We do not always receive medication at the clinic. We become so disappointed and we don’t understand why the clinic is operating without medication” (Participant 14)

#### Theme 6. The impact of an adolescent repeat pregnancy

Nine of the participants had a repeat pregnancy during their adolescent years. This theme exposed some effects of a repeat pregnancy on these adolescent mothers’ lives. The difficulties in motherhood was ascribed to the number of children born to the adolescent. The subsequent birth of a second child resulted in financial pressures.

##### Subthemes

**Increased financial hardship**

For some adolescent mothers, a repeat pregnancy compounded their financial problems. According to Participant 15, the repeat pregnancy also impacted on the financial position of her family.

“I need more money to raise two children. Both my children are still using diapers. The financial hardship has definitely increased. I am also owing people money.” (Participant 3)

“My adolescent repeat pregnancy has resulted in a financial burden for me and my grandmother. I need more financial assistance to raise my children. The financial problems do get worse for the entire family when a second baby comes unexpectedly” (Participant 15)

**Increased feelings of social isolation**

For some adolescent mothers, a repeat pregnancy resulted in increased feelings of isolation. The birth of the second child resulted in more parenting responsibilities for the adolescent mothers.

***“***I am much lonelier and secluded. I have no time for socialisation. The time I have is only for both my children” (Participant 17).

“My repeat pregnancy has made life very challenging. I feel secluded. I don’t have a social life. I miss my maternal mother who passed away because I have no emotional support (Participant 18).

**Physically and emotionally draining**

According to adolescent mothers, the repeat pregnancy and the birth of a second child can be physically and emotionally draining. Participant 2 verbalized that raising two young children can be overwhelming and tiring.

“I am physically and emotionally tired every day. I find it hard to balance my time between both children. They are both young and require my attention” (Participant 1)

“I have sleepless nights. I am tired even in the mornings. My children are both demanding of my time. I am emotionally overwhelmed with the responsibility of two children” (Participant 2)

**Guilt (conflict in spending time equally with both children)**

Adolescent repeat mothers experienced feelings of guilt because they felt that they were not spending equal time with both of their children. Their attention to their children became divided. Participant 4 felt that she had less time for her older child because she spends more time with the new baby.

“I feel like my eldest child is being neglected. I feel guilty as a mother. I leave him to play by himself sometimes because my second child is young.” (Participant 3)

“I feel that my repeat pregnancy has resulted in me having less time for my older child. I have time constraints. I feel that sometimes I forget that I have two children (Participant 16).

“I spend more time taking care of the new baby. This makes me sad that my eldest child doesn’t get much attention” (Participant 4)

#### Theme 7. Needs of adolescent mothers

Within this theme, participants expressed the following needs: financial support and independence, educational attainment, family support and support groups.

##### Subtheme

**Financial support and independence**

Adolescent mothers expressed the need for financial support and the financial independence. For Participant 15, financial independence would help ease the financial stress and burden of her family.

“I want to be financially independent so that I can secure my future” (Participant 6)

“As a young mother, I need to become financially independent so that I can take care of my children and end my grandmother’s financial burdens” (Participant 15).

“I need more financial support because my mother’s grant money is not enough to meet my children’s needs” (Participant 16)

**Educational attainment**

Most adolescent mothers voiced their need to complete their high school education.

“I need to return to school and complete my matric. I also need emotional and financial support” (Participant 11)

“My greatest wish is to complete my matric and I need to go back to school (Participant 17)

“I dropped out of school after falling pregnant. I need to return to school as I want to complete my schooling. I need to also attend university” (Participant 14)

“I need to complete high school and focus on tertiary studies and finding a job” (Participant 7)

“I need to finish my matric. I need a bursary to study further” (Participant 1)

“I need to improve myself academically by completing school and attending university” (Participant 9)

**Family support**

Adolescent mothers expressed the need for family support, encouragement, nurture, forgiveness and acceptance.

“I need the emotional and financial support from my family. Family support is important because adolescent motherhood can be difficult” (Participant 8)

“I want more emotional support from my family. They are still angry with me because I fell pregnant at a young age. I really need their support in order to progress in life” (Participant 10)

“I wish my family were more supportive. Their anger and resentment towards me has not subsided. I crave their affection towards me and my children” (Participant 3)

“I want to apologise to my parents. I want their forgiveness. I want their emotional and financial support” (Participant 12)

**Support groups**

For some adolescent mothers, a support group could fill the need for emotional support. Maya viewed the support group as an opportunity to share personal experiences and feelings with peers who have similar life experiences.

“I need a support group that can help me cope emotionally. The loss of my mother is like a vacuum in my life” (Participant 18)

“I need a support group that helps adolescent mothers. I really need the motivation” (Participant 4)

“I wish that I could talk to other adolescent mothers about my problems. I think I need a peer support group. It is difficult to open up to other peers who have not been in your shoes” (Participant 15)

**Health related information**

In this study adolescent mothers acknowledged that health related information was an important need for them. They expressed the need for information on postnatal depression, child development, nutrition, the use and effects of traditional medication to treat childhood illnesses.

Adolescent mothers indicated that they had heard about postnatal depression but they did not understand the condition. They desired more information on postnatal depression from healthcare providers.

“I read in pregnancy magazines about postnatal depression. I still don’t understand postnatal depression that well. I wish the nurses could teach us more about this condition because it affects women who have given birth” (Participant 15)

“I like to know about postnatal depression. Nobody explained this condition to me and how it would affect me and my baby” (Participant 1).

“I want more information on postnatal depression” (Participant 6)

For some adolescent mothers, information on child development was essential. Adolescent mothers were concerned about their child’s development.

“I would like more information on child development” (Participant 14)

“I would like more information on how to tell if my baby is growing well (Participant 4)

“My child’s development is important and I need information about normal child development” (Participant 8).

Adolescent mothers also expressed the need for information on child nutrition. Participant 10 had reported that her baby was diagnosed as underweight and nutritional advice was needed to ensure that she could care better for her child.

“I would like more information about healthy eating and weight gain for the baby” (Participant 3)

“I am interested in nutritional advice so that I can give my baby healthy food to eat. The nurse said my baby is underweight” (Participant 10)

For some adolescent mothers, the use of traditional medication for childhood illnesses were advocated by their elders and neighbours*.* The adolescent mothers were unsure of the safety of the traditional medications.

“The elders give us advise to use traditional medication for our babies. I would like the nurses and doctors to advise us if traditional medication is safe.” (Participant 11)

“I want to know if I can use traditional medication to treat my baby’s fever” (Participant 13)

“Sometimes there is no medication at the clinics. My neighbours advise me to use traditional medication because they say it is more efficient. I want to know if traditional medication is safe for my baby” (Participant 12)

#### Theme 8. Dreams and future aspirations

The participants admitted that they had dreams and aspirations for the future. There was an overwhelming thirst for education, to have a good career, and to be a person who contributes to society.

##### Subthemes

**Education and career**

Adolescent mothers spoke about their future career aspirations. For some adolescent mothers, a career meant a secure future and financial independence.

“I want to complete high school and obtain a matric certificate. I will attend university to study towards a social worker degree (Participant 14)

“I need to find a cooking school to provide the skills that I need to become a chef” (Participant 16).

“I want to become a successful lawyer. I want to give my child the life that I could not have” (Participant 11)

“I want to become a teacher and be financially independent” (Participant 17)

“I have a great interest in food and nutrition. I want to become a dietician” (Participant 13)

“I want to go back to school so that I can complete my matric. I will then study to become a nurse” (Participant 3)

**Contributing members of society**

Some adolescent mothers aspired to contribute positively to their community and society. Participant 15 aspires to show society that adolescent mothers have dreams and can be contributing members of society.

“My dream is to become a medical doctor and help my community” (Participant 2)

“I want to prove to society that adolescent mothers also have dreams. My dream is to become a social worker and help educate young girls to make better choices in life than I did. I want to be able to serve my community and make my grandmother proud of me” (Participant 15)

## Discussion

The aim of this study was to explore adolescent pregnancy and motherhood through the eyes of affected young women and to develop an understanding of this phenomenon and the aspirations of these mothers. The themes that emerged most predominantly were: the complexities of adolescent pregnancy; participants’ relationship with the father of the child; the prevalence of father-child interaction; the reality of life since the pregnancy and its ensuing motherhood; sources of financial and emotional support; problems experienced by adolescent mothers; needs of adolescent mothers; the impact of an adolescent repeat pregnancy; and the dreams and aspirations of adolescent mothers for a better future.

The most prevalent problems of these adolescent mothers were that they experienced financial constraints, had difficulties accessing healthcare services, discrimination and stigmatisation by healthcare workers and exposure to a judgmental society. The needs that adolescent mothers’ prioritised were: financial support and independence, educational attainment, family support, support groups, and health related information.

The participants explained that the complexities of an adolescent pregnancy included dealing with an unplanned pregnancy, being compelled to fall pregnant, their partners’ negative reaction to their pregnancy, the family’s reaction to the pregnancy, and psychological issues. It was found that unplanned pregnancies impacted their lives quite drastically. Prior studies also noted that many adolescent pregnancies were unintended [9, 13, 25–27]. An important finding was that some young women were victims of imposed pregnancies, which is a phenomenon that is prevalent in South Africa due to societal pressure [28], particularly as some men need to ‘prove’ their fertility [29]. In sub-Saharan Africa, childbearing and motherhood are regarded as integral parts of a woman’s life that it is her duty [30].

Some participants in the current study reported that their partners had desired and insisted on the pregnancy. Such pregnancies were also reported by Mohammadi et al. [31], who found this practice prevalent among married adolescent Iranian women. Mashala et al. [26] and Ntinda et al. [32] suggest that adolescents may justify their pregnancies by assigning complete responsibility to their partners. Another possible explanation for these young women’s willingness to submit to an imposed pregnancy may be attributed to the patriarchal nature of the South African society where male partners still influence the sexual and reproductive decisions of women [9, 13].

Some partners of the participants welcomed the pregnancy while others were in denial of their complicity in the pregnancy. Gyesaw and Ankomah [8] also found that partners’ reactions to a pregnancy could be diverse. Previous studies also found that the disclosure of an adolescent pregnancy could upset family members and result in strained relationships [26, 32]. The participants in our study reported that their fathers were not supportive while their mothers were more supportive once they had grown used to the idea. Strained father-daughter relationships following the disclosure of an adolescent pregnancy were found in South Africa and Swaziland [26, 32]. The revelation of suicidal ideation, anger, guilt and shame confirms that pregnant adolescents experience psychological distress. One study suggests that the risk of suicide is three times greater in pregnant adolescents than in adult pregnant women [33]. Moreover, suicidal behaviour is associated with depression, anxiety disorder, physical abuse, and low education levels [33].

The participants’ relationship with the father of the child and father-child interactions were also explored in the current study. The relationships were varied as the fathers of the children were either caring and supportive, abusive, or in denial. Some participants described their relationship with the father of the child as complicated while others’ stated the relationship was non-existing. Studies have suggested that when adolescent mothers are no longer involved in a relationship with the father of the child, the risk of the father not being involved in the child’s life is great [34, 35]. The inability of the father to contribute financially towards the care of the child also leads to the failure of the relationship and the prevalence of the ‘absent father’ phenomenon [34]. Furthermore, the denial of paternity leads to the non-involvement of the father in the child’s life [9]. Our study showed that the actively involved father was financially supportive.

The findings under the subtheme ‘it’s complicated’ suggest that an adolescent woman may have to tolerate the fact that her partner is in a relationship with another woman. Gender power imbalances and cheating in relationships have been reported by several authors [36–40]. However, the tolerance of adolescent women of their promiscuous partners renders them vulnerable to HIV/AIDS and STDs [41, 42]. Some participants’ comments about the abusive attitude of their partners also resonate with the literature. Wood and Barter [43] state that adolescent pregnancy and motherhood increases the risk of physical, sexual and emotional violence in relationships and it has been reported that South African women experience high levels of partner abuse or intimate partner violence [41–46]. Adolescent women who are young and submissive are thus at great risk of intimate partner violence [43].

The theme that emerged regarding the reality of life after pregnancy and the onset motherhood revealed that the participants felt that their children’s needs were their first priority. They struggled with a non-existent social life, loneliness, disruption of schooling, transitioning into roles of being a provider and nurturer, parenting concerns, anxiety and stress but their children’s needs came first. Leese [4] also found that adolescent mothers realised that their children’s needs had to be put before their own. Loneliness, disruption of schooling, the lack of a social life, parenting concerns, anxiety and stress are common in the lives of adolescent mothers [10, 26, 47, 48], while an unsupportive family and a judgmental society also fuel loneliness and depression in adolescent mothers [49, 50].

Clearly, the transition to motherhood can be stressful for adolescents [10, 51], but this study found that they associated their parental responsibilities with nurturing their children. Similarly, adolescent mothers in Swaziland felt that becoming a parent gave them a sense of maturity and the responsibility to provide and care for a child [32]. Ngum Chi Watts et al. [30] argue that, while motherhood brings a lot of responsibilities, African Australian adolescent mothers associated motherhood with a sense of purpose. For many of these mothers, the health and well-being of their children was a primary concern. Melvin and Uzoma [52] found that adolescent mothers in Nigeria regarded child illness as a dreadful condition. According to the current study, additional parenting concerns included obtaining baby consumables such as food and nappies, and securing the future of their children. Being concerned about the future of their children mirrored the fear that adolescent mothers in Swaziland and Nigeria expressed [32, 52].

The problems listed by the adolescent mothers also reflected their needs. Financial hardship was a primary concern, and this was consistent with similar findings by Mashala et al. [26] and Bhana and Nkani [9]. Most participants in the current study indicated that financial constraints, parenting responsibilities and a lack of their families’ support resulted in their inability to return to school. This is underscored in South African literature that states that only one third of adolescent mothers return to school [53]. In the current study, it was the participants with repeat pregnancies in particular who associated this phenomenon with dire financial hardship, an increased feeling of isolation, guilt, and physical and emotional draining. This suggests that adolescent repeat pregnancies limit educational attainment and compound the socio-economic plight of these young women [21]. A repeat pregnancy during adolescence also increases parenting stress and child neglect [21, 54], and thus adolescent repeat mothers are at high risk of depression, anxiety and suicidal ideation [21, 55].

Similar to the views expressed by adolescent mothers in Uganda [56], all the participants emphasised their need of financial support, independence, and educational attainment. Devito [47] and Vincent [57] comment that adolescent mothers perceive educational attainment as a highly valued goal. The participants also valued family support and the assistance of support groups, particularly as family support is a springboard for assisting them in the attainment of education and financial independence. This correlates with a study by Mulherin and Johnstone [58], whose participants argued that family support would ease their transition to motherhood. The literature suggests that a lack of social support results in anger and punitive parenting behaviour among adolescent mothers [59], and thus the influence of social support on the health of children should not be underestimated. Adolescent mothers who participated in a study by de Jonge [60] in Edinburgh also advocated for support groups. The benefits of support groups include improved self-esteem, improved parenting, and improved communication skills [60].

The negative perceptions that society holds of adolescent mothers was lamented by the participants in the current study. They felt that they were treated as outcasts, strangers and contagious agents. The community would accuse them of being bad role models for younger girls and they found the discrimination and stereotyping they experienced overwhelming. Ngum Chi Watts et al. [30] also found that African Australian adolescent mothers experienced exclusion, rejection and disapproval in their communities.

Societal disengagement with adolescent mothers may occur due to the perception that adolescent childbearing is shameful and the personal failure of these young women [32]. Political discourse in the United States of America and the United Kingdom has generated the view that adolescent pregnancy is a catastrophe and that adolescent mothers are unsuccessful individuals [61]. A similar discourse has plagued South Africa after former President Zuma publicly commented that adolescent mothers should be separated from their children and be sent to Robben Island [62], which served an infamous jail for political prisoners during the apartheid years. It is undeniable that negative social evaluations of adolescent pregnancy and motherhood impact the psychosocial well-being of young mothers. Adolescent pregnancy and motherhood is a topic that has been surrounded by moral, political and economic discourse [61], and all the findings discussed above clearly illuminate the controversies associated with this phenomenon. In this context, statements such as the one uttered by the former president intensify the danger of widespread moral opposition to adolescent pregnancy and motherhood [62].

Amongst all the dire consequences of adolescent pregnancy, financial resources appear to be the most prominent. The participants reported that their grandmothers, biological mothers, the paternal grandmother of the child and their partners provided financial and emotional support to various degrees. Some emphasised that their grandmothers were a positive influence in their lives. Similarly, African American grandmothers play a very important role in supporting parenting adolescent women [63]. A study by Wahn et al. [64] that was conducted in Sweden revealed that adolescent mothers regarded their own mothers as their saviours. Adolescent mothers in Swaziland also turned to their biological mothers for emotional and financial support [32]. This illustrates the importance of the mother-daughter relationship. Mothers of parenting adolescent women are a source of care, financial support and mentoring [32]. Sumo et al. [65] argue that adolescent mothers who have a difficult relationship with their own mothers are at high risk of depression. This situation can be detrimental to the well-being of these mothers and their children. Research has also shown that maternal grandparents often refer to the infant as the agent that connects the families [56]. Grandparents are generally sincere in their efforts to take responsibility for the infant as they view the infant as innocent and not responsible for the adolescent pregnancy [66].

Little is known about paternal grandmothers’ support for adolescent mothers and their children. Studies conducted by Van Zyl et al. [67] and Malindi [68] in South Africa noted that most adolescent mothers viewed their partners’ parents as a positive source of support. Some participants in the current study also found that their partners’ parents (particularly the mothers) were emotionally and financially supportive. Our study also revealed that the partners of the adolescent mothers provided emotional and financial support. This reinforces a finding by Chideya and Williams [69] that South African adolescent fathers are generally willing to nurture, provide for, and act as role models for their children. Fathers in the latter study acknowledged the financial assistance provided by their parents in caring for their children [69].

The theme of healthcare service problems suggests that long distances and transportation costs, unsympathetic nursing staff, mistreatment, and limited stocks of medication impact young mothers. A growing body of literature has also revealed that barriers in accessing maternal and child healthcare services include distant geographical locations, long travelling periods, expensive transportation, and healthcare workers’ negative attitudes [70–73]. Limited stocks of medication (or stock-out) has particularly been noted in several South African health districts [74]. Sumankurro [70] argues that running out of medication is a barrier to optimal maternal and child healthcare services in low and middle income countries.

The finding that nursing staff demonstrated a lack of empathy towards pregnant and parenting adolescent mothers is supported by earlier South African studies [21, 29, 67]. Various participants described healthcare providers as disapproving and rude and they also felt mistreated by them. A study by Govender et al. [21] among nurses in KwaZulu-Natal revealed that even nurses themselves acknowledged a lack of empathy, discriminatory attitudes, and mistreatment of adolescent mothers by their colleagues.

The participants communicated that they needed healthcare information on postpartum depression, child development, nutrition, and the effects of using traditional medication to treat their children’s illnesses. A study conducted in Thailand by Erfina et al. [75] also noted that adolescent mothers wanted to improve their knowledge of child nutrition, child growth and development, and infectious and chronic childhood diseases. Many South African nurses have insisted that health care workers should embrace the importance of parenting education for adolescent mothers [21], particularly as they are highly likely to experience challenges with childrearing and postpartum depression. Depression has a negative impact on maternal health, foetal development and mother-infant bonding. A study by Hanley and Long [76] in a semi-rural part of South West Wales among young mothers who had been diagnosed with depression found that these mothers had little prior knowledge of the condition. In this regard, maternal health education should also encompass mental health information.

In South Africa, attention has recently focused on the concept of medical pluralism which occurs when individuals utilise more than one medical system when seeking healthcare [77]. Such healthcare systems include traditional and faith-based systems. It was evident in this study that many participants had been consulting traditional healers and using or contemplated using traditional medication. In Africa, 80% of the population reportedly use traditional medication, particularly in rural African communities, and the prevalence of the use of traditional medication for maternal and reproductive health issues ranges from 21 to 79.9% on this continent [78]. According to Shewamene et al. [78], traditional medications is commonly associated with a lack of formal education and low income. In the South African context, referring children to traditional healers is common [79]. In fact, traditional healers are sometimes consulted before a medical doctor [80]. Prompted by high rates of herbal intoxication in children, a study by Dambisya and Tindimwebwa [79] in the Eastern Cape among mothers and caregivers found that 57.3% of the respondents had used traditional remedies in the treatment of their children. Disconcertingly, statistics from the peadiatric department at the Umtata General Hospital in the Eastern Cape revealed that 40% of children admitted for herbal intoxication had died [79]. Herbal intoxication can cause severe liver and kidney damage [79]. The fact that the participants requested more information on the effects of using traditional medications is thus warranted.

On a positive note, the participants in this study shared their dreams and aspirations for the future. They envisioned completing their education and focusing on their dream careers and some verbalised the hope of contributing meaningfully to society [58, 81]. Previous studies have shown that adolescent mothers can be resilient and that they can persevere to achieve a brighter future for themselves and their children, and most consider that their future is interconnected with that of their children. A study by O’Brien Cherry et al. [81] in Indiana in the US found that all 52 of the participating adolescent mothers aspired to provide a worthy future for their children by completing school and following a career. Adolescent mothers who had persevered through high school attributed their success to hope, confidence, determination, self-assurance, encouragement, intrinsic and extrinsic motivation, respect, and support from their teachers, alternative classes, the community, life skills programmes, and their peers [81].

### Strengths and limitations

Traditionally, studies on adolescent pregnancies focused on the experiences of first time adolescent mothers. The strength of this study can be attributed to the inclusion of both first time and repeat mothers. However, the study was confined to female participants only. It is acknowledged that adolescent pregnancy and parenting is not a gender specific problem and that the experiences of adolescent men also have important implications for health service delivery. Moreover, the study utilised only a few participants (*n* = 18) and this resulted in only four focus group discussions. Another limitation is that the study was limited to one health facility in one provincial district. However, the adolescent delivery rate is high at this facility. In light of the limitations, the findings of this study may not be generalised and can be transferred only to a similar research setting.

## Conclusion

Based on the findings, it was concluded that experiences of adolescent pregnancy and parenting are multifaceted. This argument is corroborated by Govender et al. [21], who insists that adolescent mothers and their children require a multidisciplinary approach to healthcare. While researchers and policy makers have often viewed adolescent pregnancy and parenting as catastrophic and a societal failure, this study suggests that adolescent mothers envision a better future for themselves and their children, and the authors thus propose that support strategies should be strengthened to guide them on this journey. The findings enhanced our understanding of the medical and psychosocial barriers that stifle the future that adolescent mothers desire, and it was clearly illuminated that the healthcare needs of pregnant and parenting adolescents extend beyond information and knowledge about giving birth. A collation of evidence suggests that interventions that focus on comprehensive medical services, health education, health promotion, psychosocial interventions and school enrolment will have positive outcomes for adolescent mothers and their children [6]. Thus a key policy priority is the collaboration of various professionals from a variety of related sectors to assist adolescent mothers in achieving better health and psychosocial and socio-economic outcomes and to guide them on their way to a better future.

## Data Availability

The data used to elicit the findings of this study are available from the corresponding author upon reasonable request.

## Abbreviations

HIV: Human immunodeficiency virus
AIDS: Acquired immunodeficiency syndrome
SRH: Sexual and reproductive health
STI: Sexually transmitted disease
